# Galantamine mitigates testicular injury and disturbed spermatogenesis in adjuvant arthritic rats via modulating apoptosis, inflammatory signals, and IL-6/JAK/STAT3/SOCS3 signaling

**DOI:** 10.1007/s10787-023-01268-z

**Published:** 2023-07-10

**Authors:** Sara I. Shafiey, Kawkab A. Ahmed, Ali A. Abo-Saif, Amira M. Abo-Youssef, Wafaa R. Mohamed

**Affiliations:** 1 Department of Pharmacology and Toxicology, Faculty of Pharmacy, Nahda University, Beni-Suef, 62514 Egypt; 2 Department of Pathology, Faculty of Veterinary Medicine, Cairo University, Giza, 12211 Egypt; 3 Department of Pharmacology and Toxicology, Faculty of Pharmacy, Beni-Suef University, Beni-Suef, 62514 Egypt

**Keywords:** Galantamine, CFA, Rheumatoid arthritis, Testicular injury, Apoptosis, IL-6/JAK/STAT3/SOCS3

## Abstract

Rheumatoid arthritis (RA) affects the joints and the endocrine system via persistent immune system activation. RA patients have a higher frequency of testicular dysfunction, impotence, and decreased libido. This investigation aimed to evaluate the efficacy of galantamine (GAL) on testicular injury secondary to RA. Rats were allocated into four groups: control, GAL (2 mg/kg/day, p.o), CFA (0.3 mg/kg, s.c), and CFA + GAL. Testicular injury indicators, such as testosterone level, sperm count, and gonadosomatic index, were evaluated. Inflammatory indicators, such as interleukin-6 (IL-6), *p*-Nuclear factor kappa B (NF-κB p65), and anti-inflammatory cytokine interleukin-10 (IL-10), were assessed. Cleaved caspase-3 expression was immunohistochemically investigated. Protein expressions of Janus kinase (JAK), signal transducers and activators of transcription (STAT3), and Suppressors of Cytokine Signaling 3 (SOCS3) were examined by Western blot analysis. Results show that serum testosterone, sperm count, and gonadosomatic index were increased significantly by GAL. Additionally, GAL significantly diminished testicular IL-6 while improved IL-10 expression relative to CFA group. Furthermore, GAL attenuated testicular histopathological abnormalities by CFA and downregulated cleaved caspase-3 and NF-κB p65 expressions. It also downregulated JAK/STAT3 cascade with SOCS3 upregulation. In conclusion, GAL has potential protective effects on testicular damage secondary to RA via counteracting testicular inflammation, apoptosis, and inhibiting IL-6/JAK/STAT3/SOCS3 signaling.

**Graphical abstract:**

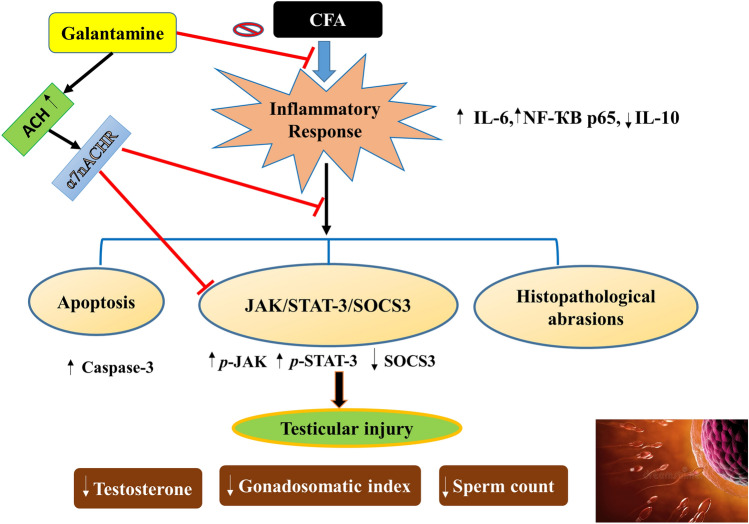

## Introduction

Rheumatoid arthritis (RA) is a progressive inflammatory disorder that affects the joints by persistent immune system activation. Various environmental and genetic factors play a central role in RA pathogenesis (Choy 2012). Joint damage in RA begins with macrophage infiltration and CD4 + T lymphocyte activation inducing various cytokines, such as interleukin-6 (IL-6), interleukin-1 (IL-1), tumor necrosis factor-alpha (TNF-α), nuclear factor kappa (NF-ҡB), and free radicals, which cause synovial membrane hyperplasia and increased vascularization (Hemshekhar et al. 2012; Ostrowska et al. 2018).

Inflammatory mediators from the swollen inflamed joint induce extra-articular tissue injuries including testicular impairment, disruption of the spermatogenesis process, and impotence (Bove 2013; Cojocaru et al. 2010). RA patients were found to suffer from hypogonadism with reduced testosterone which decreases libido and affects reproduction (Karagiannis and Harsoulis 2005). Reduced testosterone levels are attributed to the activation of macrophages in testes by arthritis (Bendele et al. 1999; Santos et al. 2020). On the other hand, it has also been noted that men with untreated hypogonadism have an increased frequency of autoimmune disorders, suggesting that testosterone exerts an immunosuppressive effect (Brubaker et al. 2018). RA can also have an impact on the male sex glands through affecting both the growth of the epithelium and the secretory activity of these glands (Toivanen and Shen 2017).

Complete Freund’s adjuvant (CFA), heat-killed mycobacterium, is frequently used for the experimental induction of RA due to its powerful immune stimulatory impact (Billiau and Matthys 2001). CFA was reported to induce testicular impairment through stimulating numerous innate immune system cells, enhancing endogenous inflammatory cytokines expression and testicular macrophages, which has been shown to suppress testicular androgen synthesis, lower blood testosterone bioavailability, and undermine testicular steroidogenesis (Xiao et al. 2018). This model resembles testicular damage in RA patients and has been used frequently for studying testicular damage pathophysiology and mechanisms secondary to RA in animals (Clemens and Bruot 1989; Darwish et al. 2014; Santos et al. 2020).

Inflammation has been characterized as a vital player in CFA-induced testicular injury through sperm DNA damage, inducing germ cell apoptosis, and spermatogenesis impairment (Hassan et al. 2019). Previous research reported a significant increase of IL-6 in testicular tissue with CFA (Musha et al. 2013). Binding of IL-6 to the Janus kinase (JAK)/signal transducers and activators of transcription3 (STAT3) receptor family induces phosphorylation of STAT3 with subsequent nuclear translocation and stimulation of several inflammatory and apoptotic genes transcription including NF-κB (Cha et al. 2015; Fouad et al. 2020). Suppressor of cytokine signaling 3 (SOCS3) is the most significant member of the SOCS family, as it can inhibit JAK/STAT3 signaling pathway in response to stimuli, such as cytokine, growth factors, and mitosis (Xiao et al. 2018). SOCS3 limits binding of JAK kinase inducing suppression of JAK phosphorylation and competes with JAK to inhibit STAT3 phosphorylation (Lin et al. 2010). Therefore, modulation of IL-6/JAK/STAT3/SOCS3 signaling could be an essential strategy for the treatment of CFA-induced testicular injury.

Apoptosis of spermatids and spermatocytes is an essential feature that indicates seminiferous tubule destruction in RA (Jacobo et al. 2011). The anti-apoptotic B-cell lymphoma-2 (Bcl-2), pro-apoptotic Bcl-2-associated-X-protein (Bax), and caspase proteins control the balance between cell proliferation and apoptosis. Induction of Bax proteins induces cytochrome c and other apoptogenic substances, resulting in the formation of apoptosomes, which then induce caspase-3-dependent cell death (Xiao et al. 2018). Apoptosis maintains the balance between germ cells and Sertoli cells. Sertoli cells are crucial for the development of germ cells, from the maintenance of the spermatogonial stem cell niche through meiosis and spermatogenesis to the release of fully developed spermatids during spermiation. Male infertility has been connected to an imbalance in this mechanism (Crisóstomo et al. 2018).

Acetylcholine is highly expressed in the Leydig and Sertoli cells of the testes, where its actions include increased vasoactivity, sperm moving through the excurrent duct system, cell secretion, muscle contraction, and cell proliferation within the sex accessory glands (Christina et al. 2010). It has an essential function in inflammation as it motivates vagus nerve leading to decreased cytokine production via stimulation of α-7 nicotinic acetylcholine receptor (Liu et al. 2010). From a physiological perspective, serum acetylcholine esterase (ACHE) activity may harm the testes. It was proved that AChE action increases with RA and could be involved in testicular impairment through inducing inflammatory responses (Ofek et al. 2007). Inhibitors of ACHE (ACHEIs) were proven to exert potential anti-rheumatic effect (Gowayed et al. 2020; Kandil et al. 2020) and could have a role in the management of testicular impairment secondary to RA providing single treatment for RA patients suffering from testicular impairment.

Galantamine (GAL) is a member of ACHEIs that is clinically used for management of Alzheimer's disease and was reported to possess potent anti-inflammatory and immune-modifying properties (Nizri et al. 2005, 2006). Previous reports demonstrated that GAL has anti-inflammatory effects through the reduction of pro-inflammatory cytokines (TNF-α) in a model of autoimmune encephalomyelitis (El-Emam et al. 2021) and increased IL-10 in a model of RA (Gowayed et al. 2015). Additionally, GAL reduced JAK/STAT3 expression in acute kidney injury (AKI) model through modulation of NF-κB (p65) and IL-6/JAK2/STAT3/SOCS3 signals via α7nAChR (Ibrahim et al. 2018) and in experimental asthma model, α7nAChR exerted promising effects by inhibiting NF-κB/STAT3/SOCS3 signaling. Interestingly, GAL has shown a potential anti-rheumatic effect in various studies (Bartikoski et al. 2021; Gowayed et al. 2019). Consequently, this work was designed to study the potential protective effects of GAL in the management of testicular impairment secondary to RA through targeting Caspase-3, NF-κB p65, and IL-6/JAK/STAT3/SOCS3 signaling.

## Materials and procedures

### Materials

Both CFA and GAL were purchased from Sigma-Aldrich (USA). Calbiotech (USA) provided an ELISA Kit for serum testosterone. ELISA kits for IL-10 and IL-6 assessment were purchased from Elabscience Biotechnology Inc. (USA). Santa Cruz Biotechnology (USA) provided the Caspase-3, NF-κB p65, STAT3, *p*-STAT3, JAK, *p*-JAK, and SOCS3 antibodies.

### Animals

Male adult Wistar rats (200–240 g) were purchased from the animal house at Nahda University in Beni-Suef, Egypt, at the age of six weeks. The animals had complete access to food and water in an environment with controlled humidity (60–10%), photoperiods (12/12 h), and temperature (25–2 °C). The guidelines of Beni-Suef University Institutional Animal Care and Use Committee (BSU-IACUC) with approval NO. 020-94 which follow the NIH Guidelines for Laboratory Animals Care and Use (NIH Publication updated 1985) were followed in all procedures and methods in this work.

### Experimental plan

Male adult twenty-four Wistar rats were divided into 4 groups of six rats/group as follows:

Control group: rats were administered 0.3% CMC, p.o. as vehicle daily for 15 days.

GAL group: received GAL in 0.3% CMC (2 mg/kg; p.o) daily for 15 days (de la Tremblaye et al. 2017; Njoku et al. 2019; Zeng et al. 2020a).

CFA group: On day one, 0.1 ml of CFA was subcutaneously injected into the plantar surface of the right hind paw to induce arthritis. One hour and twenty-four hours after the CFA paw vaccination, each animal received two additional booster doses each of 0.1 ml of CFA near the tail root. It should be observed that a small dose of 0.1 mg of CFA was used to induce arthritis to lower the rate of rat death, while the two identical booster doses were used to increase the impact on the systemic immune system (Arab et al. 2017).

CFA + GAL group: received CFA and GAL as prescribed above.

**Fig. 1:**
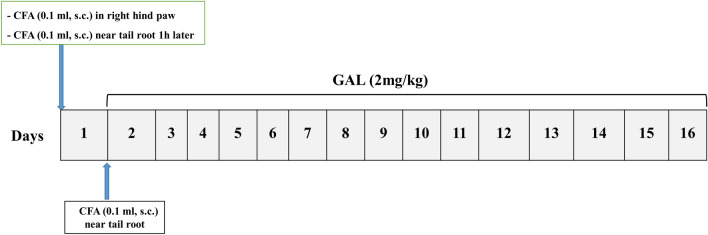
Schematic presentation of treatments protocol

Galantamine was given for fifteen consecutive days, from the 2nd to the 16th day after the CFA injection (Fig. 1).

### Serum preparation

Rats were anesthetized with 5 mg/kg xylazine and 45 mg/kg ketamine i.p (Abdel-Wahab et al. 2021) and blood was collected from the retro-orbital venous plexus, centrifuged at 3000 rpm for10 min at 4 °C and serum was separated then stored at – 80 °C for further analysis of testosterone.

### Preparation of testis

Following serum collection, a lower abdominal incision was performed to open the peritoneal cavities and the testes were exposed. Both testes and the cauda epididymis were dissected and then washed using cold phosphate-buffered saline. For histological analysis and immunohistochemical detection of caspase-3 and NF-κB p65 protein expression, one testis was kept in 10% buffered formalin. The residual testis was cut into small pieces and homogenized in phosphate buffer, centrifuged (2000 rpm, 4 °C, 20 min), and the supernatant was then separated for analysis of IL-10 and IL-6. Another section was preserved in lysis buffer for Western blot analysis of JAK, *p*-JAK, STAT3,* p*-STAT3, and SOCS3 expression.

### Biochemical investigations

#### Assay of testosterone

Following the assay kit methods, ELISA kit was used to measure serum testosterone levels (CALBIOTECH Inc., Cordell Ct., El Cajon, USA). The assay is based on the colorimetric detection of testosterone levels at 450 nm.

#### Sperm count

After separation of cauda epididymis, it was chopped into small pieces and pressed into a clean Petri dish. The semen was diluted with normal saline and viewed on a hemocytometer slide for sperm count under a microscope. The numbers in the squares were fed into a calculator for cell counting to determine the overall sperm count (Hifnawy et al. 2020).

#### Estimation of gonadosomatic index

Atrophy of the testes is a symbol of spermatogenic injury (Li et al. 2015). The gonadosomatic index was determined for each animal using the equation below (Mohamed et al. 2019):

$${\text{Gonadosomatic index}} = \left( {{\text{Weight of the 2 testes}}/{\text{Body weight}}} \right) \times {1}00.$$

#### Estimation of testicular cytokines level

According to the manufacturer’s instructions, ELISA kits were used to measure testicular IL-6 and IL-10 (Elabscience Biotechnology Inc., USA). Values were presented as pg/g.

#### Histopathological study

Testes were harvested, fixed in 10% buffered formalin, dehydrated, cleared using xylene, and embedded in paraffin. Thick slices (4–5 μm) were created, stained with Hematoxylin and Eosin (H & E) (Suvarna et al. 2018), and inspected by an experienced pathologist under a light microscope (BX43, Olympus) where the identity of specimen was kept anonymous during images capturing and analysis, so potential bias could be avoided.

The testicular scoring system was evaluated according to modified Johnsen’s scoring (El Makawy et al. 2019). These criteria are based on the evaluation of Sertoli cells and the four main spermatogenic cell types (primary spermatocytes, spermatogonial cells, spermatid cells, and secondary spermatocytes). This approach assigns a score for each seminiferous tubule in randomly selected seminiferous tubules in each group under power field × 200, between 1 (no seminiferous epithelium) and 10 (complete spermatogenesis documented).

#### Immunohistochemical study

Testicular expression of Cleaved Caspase-3 and NF-κB p65 was examined as reported previously (Fouad and Ahmed 2021). Sections from paraffin blocks were rehydrated, blocked using 5% BSA in Tris-buffered saline, and treated with primary anti-caspase-3 antibodies and anti-NF-κB p65 antibodies (Cat. # sc-7148 and sc-8008, respectively) at a 1:100 dilution for overnight incubation at 4 °C. Incubation of slides with goat anti-rabbit IgG-FITC (Cat. # sc-2012; 1:100), the matching secondary antibody was done. To demonstrate the immunological response, diaminobenzidine tetrachloride was used (DAB). The immune-positive reactive cells cytoplasm was colored brown. The degree of staining was assessed as either robust, weak, or negative (no staining). The percentage area of positive cells from 5 randomly selected fields in each section was calculated and averaged for cleaved caspase-3 and NF-κB p65 quantification using image analysis software (Image J, version 1.46a, NIH, Bethesda, MD, USA).

#### Western blot analysis

To investigate the influence of GAL on the JAK/STAT3/SOCS3 signaling, testes sections were homogenized in lysis buffer (mM Tris–HCl), pH 7.4 containing 1% protease inhibitor for 10 min at 4 °C. Bradford's technique was used to determine the total protein content of each sample (Kruger 2009). 50 µg of total protein was loaded in each lane and electrophoresed with 10% SDS–polyacrylamide gel and then transferred to a PVD membrane using a semi-dry transfer method. The membranes were blocked by 5% FBS in TBST buffer and then incubated with primary antibodies against JAK, *p*-JAK, STAT3, and *p*-STAT3, overnight at 4 °C. After rinsing membranes with TBST, they were incubated for one hour with ALP-conjugated secondary antibody. The bands were seen using the Genemed Biotechnologies BCIP/NBT Substrate Detection Kit. Image J^®^ software (National Institutes of Health, USA) was used to investigate the shaped bands concerning β-actin (Wang et al. 1996).

#### Statistical analysis

The parametric data were statistically analyzed using one-way ANOVA followed by Tukey–Kramer post hoc test which was performed to test statistical significance among experimental groups. Statistical Set for Social Sciences (SPSS version 19.0) computer software program (SPSS Inc., Chicago, IL, USA) was used. All parametric data are presented as mean ± standard error of mean (SEM).

For non-parametric data (Johnsen’s scoring), the Kruskal–Wallis test followed by the Dunn’s multiple comparison post-test was performed to test statistical significance. The non-parametric data are presented as the median and the interquartile range. Statistical significance was defined as a *p* value < 0.05.

## Results

### GAL improves serum testosterone levels in CFA-treated rats.

CFA group exhibited a significant decline in serum testosterone levels to 64.1% compared to control (*p* < 0.01). GAL elevated serum testosterone levels to about 1.43-fold compared to the CFA group (*p* < 0.01). The rats treated with GAL alone show no significant differences from control, indicating the safety of GAL **(**Table 1**).**

**Table 1:** GAL improves serum testosterone levels in CFA-treated rats

Drugs and doses	Parameters
	Testosterone (ng/ml)
Control (0.3% CMC)	7.5 ± 0.62
GAL (2 mg/kg)	7.51 ± 0.35
CFA	4.81 ± 0.35**
CFA + GAL	6.91 ± 0.45^##^

Each value represents mean ± SEM (*n* = 6)

*GAL* galantamine, *CFA* complete Freund’s adjuvant

**Significance at *p* < 0.01 versus control

^##^Significance at *p* < 0.01 versus CFA

### GAL improves sperm count and gonadosomatic index of CFA-treated rats

**Fig. 2:**
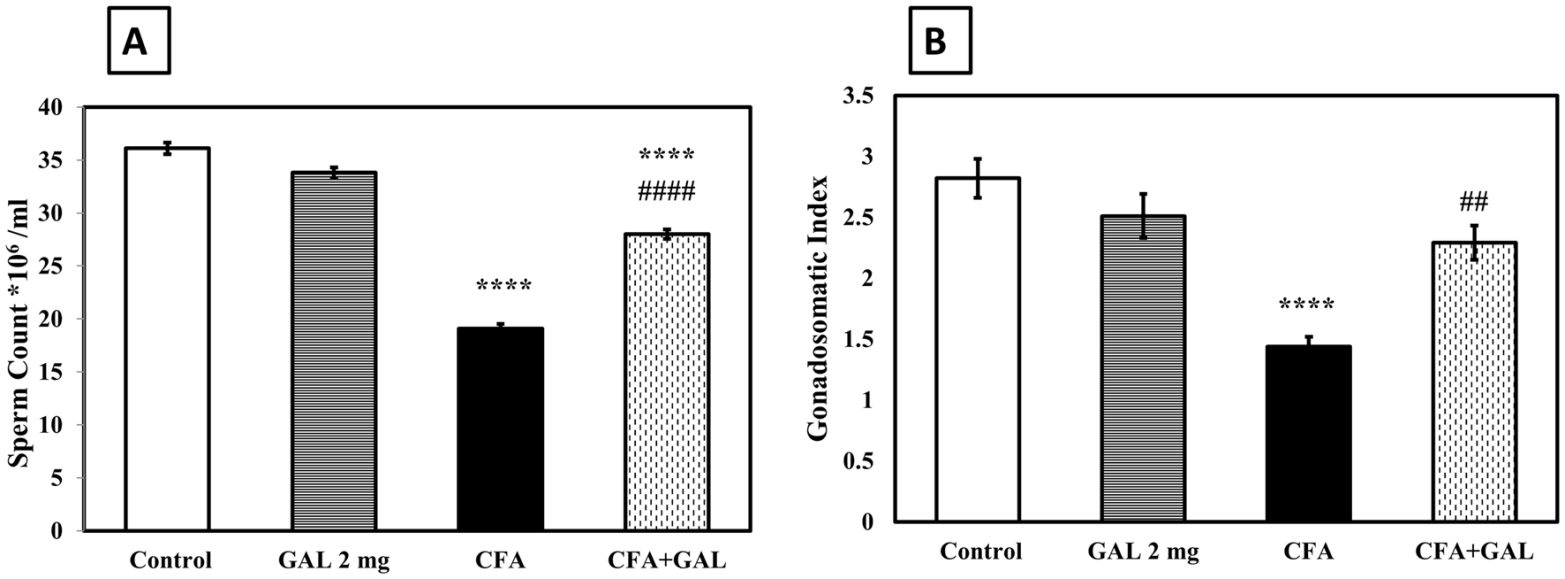
GAL improves sperm count and gonadosomatic index of CFA-treated rats. Sperm count (**A**) and gonadosomatic index (**B**) Data are presented as mean ± SEM (*n* = 6). One-way ANOVA followed by Tukey–Kramer post hoc test was performed to test statistical significance among experimental groups. ****p < 0.0001, significance from control and ^*##*^*p* < 0.01 or ^#*###*^*p* < 0.0001, significance from CFA. *GAL* galantamine, *CFA* complete Freund’s adjuvant

The potential effect of GAL against testicular injury by CFA was investigated through sperm count and gonadosomatic index measurement. The CFA group revealed a notable decline in sperm count to about 52.9% and gonadosomatic index to about 51% compared to control (*p* < 0.0001). GAL improved these alterations by returning the sperm count (*p* < 0.0001) and gonadosomatic index (*p* < 0.01) near to normal, directing its ability to attenuate RA-induced testicular injury and spermatogenesis disruption (Fig. 2).

### GAL counteracts inflammatory response in the testis of CFA-treated rats

**Fig. 3:**
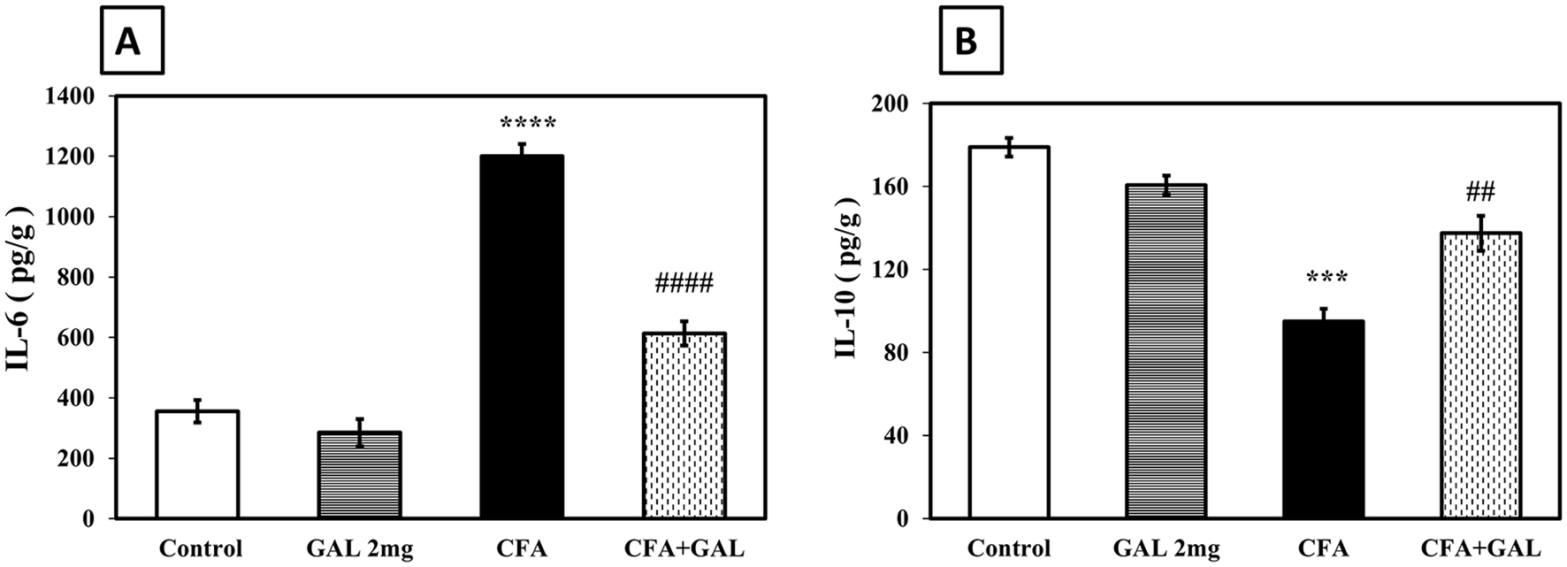
GAL counteracts inflammatory response in testis of CFA-treated rats. Tissue IL-6 (**A**) and tissue IL-10 (**B**). Data are presented as mean ± SEM (*n* = 6). One-way ANOVA followed by Tukey–Kramer post hoc test was performed to test statistical significance among experimental groups. ***p < 0.001, or ****p < 0.0001, significance from control and ^*##*^*p* < 0.01 or ^#*###*^*p* < 0.0001, significance from CFA. *IL-6* interleukin-6, *IL-10* interleukin-10, *GAL* galantamine, *CFA* complete Freund’s adjuvant

**Fig. 4:**
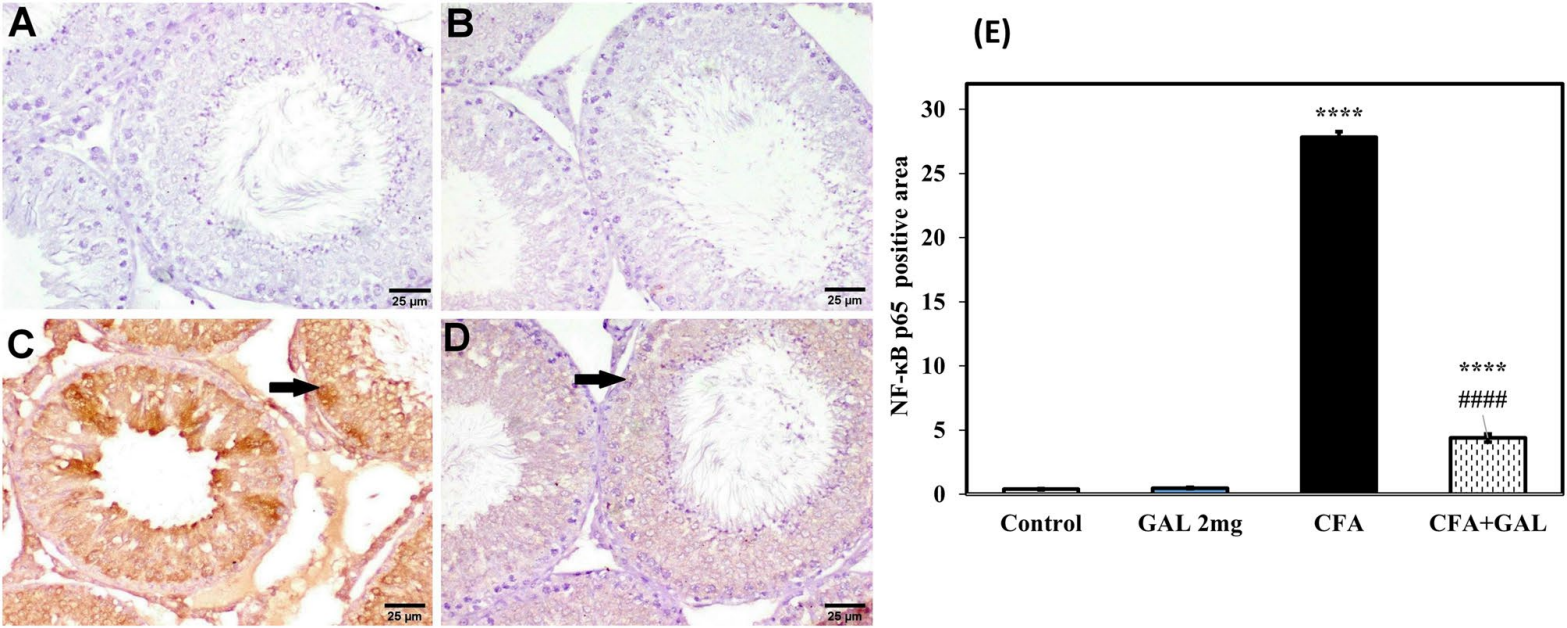
GAL reduces NF-κB p65 expression in testicular injury by CFA. Representative photomicrographs of NF-κB p65 immune expression in the testicular tissue; **A** normal control; **B** GAL control, showing negative immune expression. **C** CFA group, showing strong immunoreactivity with a significant increase of positive immunostaining cells. **D** CFA + GAL, showing weak expression of NF-κB p65 (scale bar 25 μm). **E** Image analysis of immuno-positive areas of NF-κB p65. One-way ANOVA followed by Tukey–Kramer post hoc test was performed to test statistical significance among experimental groups. ****p < 0.0001, significance from control and ^#*###*^*p* < 0.0001, significance from CFA. *GAL* galantamine, *CFA* complete Freund’s adjuvant

Rats treated with CFA exhibited a significant increase in IL-6 to 3.38-fold (*p* < 0.0001), a significant decrease in IL-10 to about 53.1% (*p* = 0.001) (Fig. 3), and strong positive expressions of NF-κB p65 (Fig. 4C) compared to control (*p* < 0.0001). Furthermore, treatment with GAL (2 mg/kg) significantly reduced IL-6 levels to 51%, increased IL-10 levels to 1.44-fold (*p* < 0.0001 and *p* < 0.01), and exhibited a weak immune reaction of NF-κB p65 compared to CFA group (Fig. 4D) (*p* < 0.0001). These observations suggest that GAL modulation of inflammatory response is involved in combating RA-related testicular impairment. GAL control group shows no significance from control concerning IL-6 and IL-10 with a negative immune expression of NF-κB p65 in the testicular tissue of control as well as GAL control rats (Figs. 3, 4A, B).

### GAL alleviates CFA-induced testicular histopathologic alterations

**Fig. 5:**
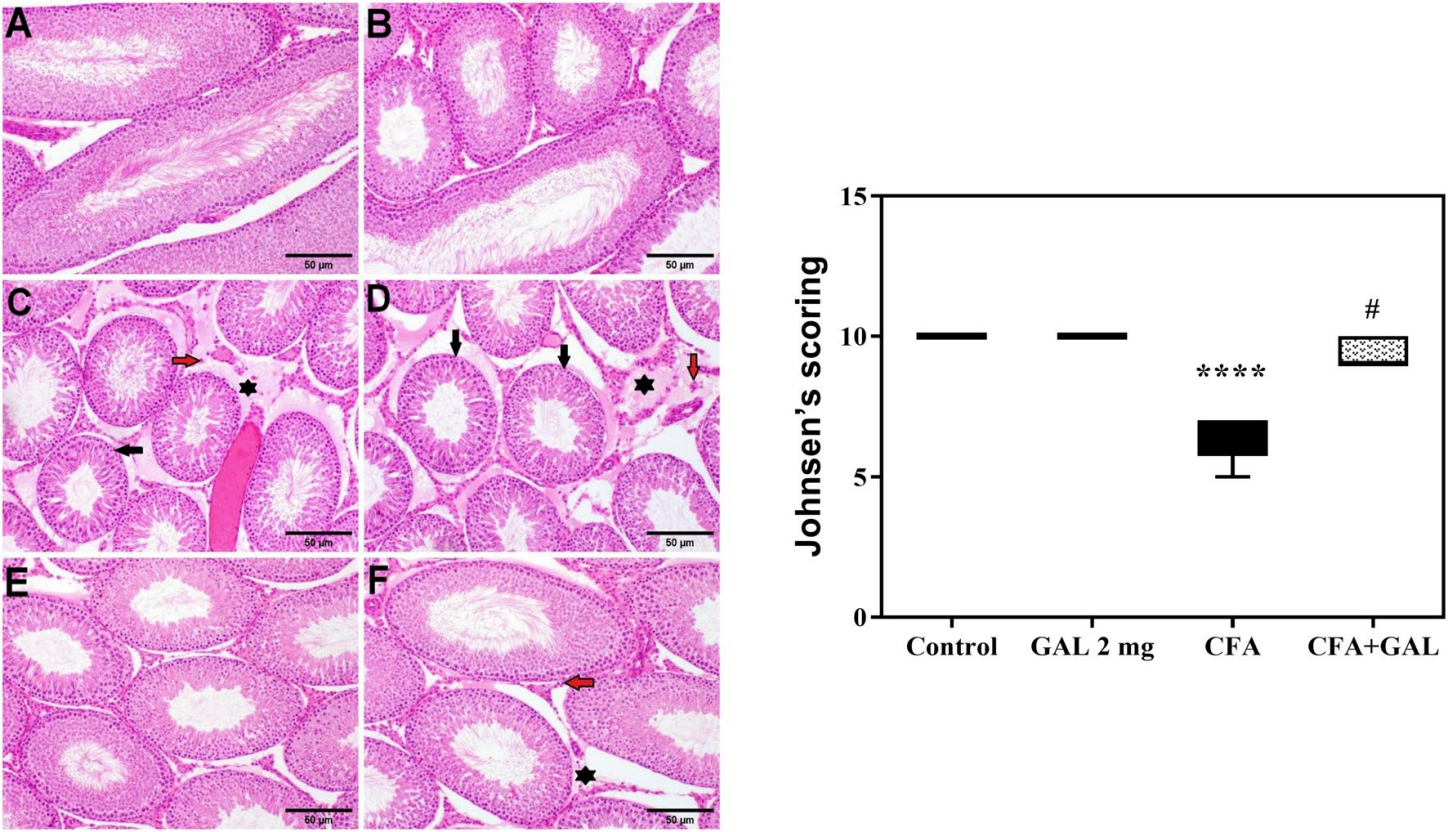
GAL alleviates CFA-induced testicular histopathologic alterations. **A** Representative photomicrographs of H & E-stained testicular tissue sections of rats; **A** normal control and **B** GAL control, showing the normal histological structure of seminiferous tubules with normal spermatogonial cells, and Sertoli and Leydig cells. **C**,** D** CFA group; showing small diameter seminiferous tubules (black arrow), marked interstitial edema (asterisk), and necrosis of Leydig cells (red arrow). **E**,** F** CFA + GAL; **E** showing re-establishment of the normal spermatogenic series, and Sertoli and Leyding cells; **F** showing mild interstitial edema (asterisk) and sparsely necrosis of Leydig cells (red arrow) (scale bar, 50 μm). Johnsen’s scoring of testicular histopathological lesions by CFA and GAL beneficial effects was presented as median and the interquartile range. The Kruskal–Wallis followed by the Dunn’s test was performed to analyze the significant difference among tested groups. ****p < 0.0001, significance from control and ^*#*^*p* < 0.05 indicates significance from CFA. *GAL* galantamine, *CFA* complete Freund’s adjuvant

Microscopically, testes from normal control rats, as well as rats treated with GAL, revealed healthy histological structure of spermatogonial cells and seminiferous tubules, Sertoli and Leydig cells with complete spermatogenesis and sperm production (Fig. 5A, B). On the contrary, rats given CFA displayed a variety of histological changes, including Leydig cell necrosis, interstitial edema, small diameter seminiferous tubules with spermatogonial cell degeneration, and congested testicular blood arteries (Fig. 5C, D). On the other hand, treatment with GAL + CFA showed observable improvement via restoration of the normal spermatogenic series, and Sertoli and Leydig cells (Fig. 5E). Some examined sections showed mild interstitial edema and sparse necrosis of Leydig cells (Fig. 5F). Furthermore, modified Johnsen’s scoring testicular lesion score was examined in the various experimental groups. CFA significantly reduced scoring (*p* < 0.0001) relative to control. Treatment with GAL significantly decreased (*p* < 0.05) the testicular lesion scoring induced in the CFA group.

### GAL counteracts apoptosis in testicular injury by CFA

**Fig. 6:**
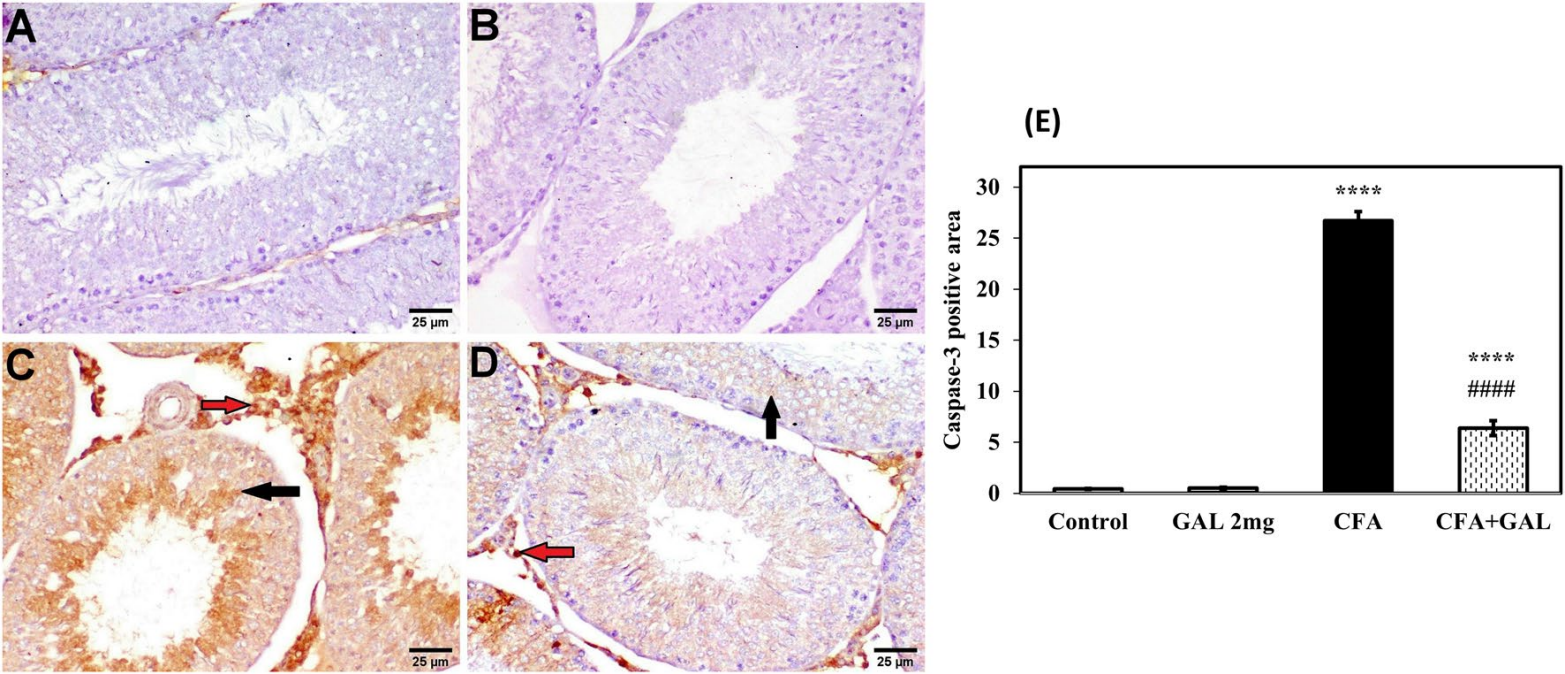
GAL counteracts apoptosis in testicular injury by CFA. Representative photomicrographs of cleaved caspase-3 immune expression in the testicular tissue. **A** Normal control; **B** GAL control, showing negative immune expression. **C** CFA group, showing strong immunoreactivity with a significant increase of positive immunostaining cells. **D** CFA + GAL, showing weak cleaved caspase-3 expression (scale bar 25 μm).** E** Image analysis of immuno-positive areas of cleaved caspase-3. One-way ANOVA followed by Tukey–Kramer post hoc test was performed to test statistical significance among experimental groups. ****p < 0.0001, significance from control and ^#*###*^*p* < 0.0001, significance from CFA. *GAL* galantamine, *CFA* complete Freund’s adjuvant

Immune-histochemical staining of cleaved caspase-3 is illustrated in Fig. 6. In brief, negative immune expression was detected in the testicular tissue of control rats as well as GAL-administered rats (Fig. 6A, B). On the contrary, significant positive expressions of cleaved caspase were exhibited by CFA group (*p* < 0.0001) (Fig. 6C, E). Otherwise, a weak immune reaction was detected in sections of GAL + CFA-treated group compared to CFA group (*p* < 0.0001) (Fig. 6D, E).

### GAL modulates JAK/STAT3/SCOCS3 signaling in rat testicular injury by CFA

**Fig. 7:**
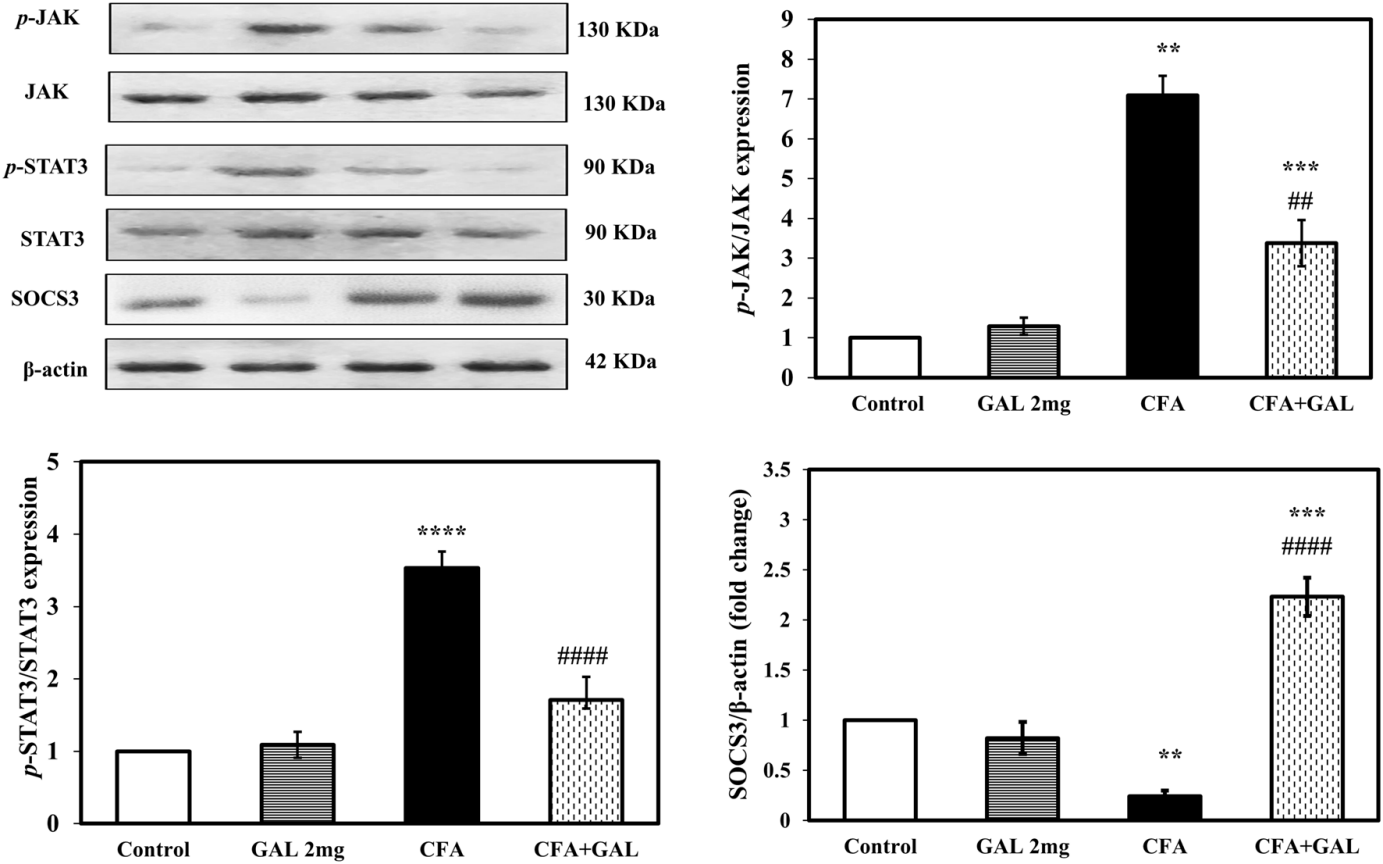
GAL modulates JAK/STAT3/SCOCS3 signaling in rat testicular injury by CFA. Results are presented as mean ± SEM (*n* = 3). One-way ANOVA followed by Tukey–Kramer post hoc test was performed to test statistical significance among experimental groups. **p < 0.01, ***p < 0.001, or ****p < 0.0001, significance from control and ^*##*^*p* < 0.01 or ^#*###*^*p* < 0.0001, significance from CFA. *GAL* galantamine, *CFA* complete Freund’s adjuvant, *JAK* Janus kinase, *STAT3* signal transducers and activators of transcription, *SOCS3* suppressors of cytokine signaling 3

Rats treated with CFA showed a significant increase in testicular JAK and STAT3 phosphorylation as proven by elevated *p*-JAK/JAK and *p*-STAT3/STAT3 ratio to about 7.09- and 3.53-fold, (*p* < 0.01 and *p* < 0.0001), respectively, and a significant decrease in SOCS3 to about 24% (*p* = 0.01) relative to control. GAL significantly decreased testicular JAK and STAT3 phosphorylation as evidenced by decreased *p*-JAK/JAK and *p*-STAT3/STAT3 ratio to 47.6% and 48.4%, (*p* < 0.01 and 0.0001) respectively, while significantly increased SOCS3 expression to about 9.2-fold relative to CFA (*p* < 0.0001). Administration of GAL to normal rats did not alter JAK, *p*-JAK, STAT3, *p*-STAT3, and SOCS3 expression compared with control (Fig. 7).

## Discussion

In the present work, we studied the ability of GAL to counteract testicular damage secondary to RA. RA is a well-known autoimmune disorder characterized by overshooting of inflammatory mediators, and oxidative and apoptotic markers that negatively affect the body function and the joints, such as the endocrine system (Bove 2013; Shen et al. 2020; Zafari et al. 2019). The most complications of RA include diminished fertility and testicular dysfunction with low levels of circulating testosterone (Karagiannis and Harsoulis 2005).

In the current study, CFA significantly lowered gonadosomatic and sperm counts compared to control. These findings were supported by the histological examination, which revealed congestion of testicular blood vessels, short dimension of seminiferous tubules and spermatogonial cells in decline, marked interstitial edema, and necrosis of Leydig cells. Early studies suggested that CFA stimulates testicular injury, reduced testosterone level, and reduced sperm count (Ahmed et al. 2021; Arab et al. 2019; Darwish et al. 2014; Eid et al. 2019). GAL successfully revoked the gonadosomatic index with an improvement in sperm count and serum testosterone level compared with CFA-treated rats. These findings demonstrate the potential effect of GAL for the first time against testicular damage and male reproductive toxicity secondary to RA.

Inflammation has a central role in testicular damage pathogenesis secondary to RA as inflammatory mediators from the joint induce extra-articular injuries including testicular damage and steroidogenesis impairment (Bove 2013; Cojocaru et al. 2010; Dutta et al. 2021). NF-κB p65 is a transcription factor that strongly links inflammation, oxidative stress, and apoptosis (Aslan et al. 2020). Under normal conditions, IκB binds to NF-κB in the cytoplasm while in response to inflammatory stimuli, IκB is phosphorylated and broken down, enabling NF-κB to move to the nucleus inducing transcription of various inflammatory cytokines including IL-6. IL-6 transcribed by NF-κB p65 exerts several effects involved in testicular injury pathogenesis, as apoptosis promotion, ROS, and inflammation induction (Arafa et al. 2020; Liang et al. 2018). IL-10 was reported to decrease pro-inflammatory cytokines, such as TNF-α and interleukins by inhibiting MHC class II antigen expression (Arafa et al. 2020). Our data revealed a marked increase in the testicular inflammatory response with elevated IL-6 and NF-κBp65 testicular expression, and reduced levels of anti-inflammatory mediator IL-10. In harmony with these results, a previous study revealed that CFA caused an increase in the expression of NF-κB p65 in models of arthritis (Abid et al. 2022; Akhter et al. 2022). Previous publications stated that CFA increased IL-6 and decreased IL-10 in a model of chronic inflammatory pain and a model of RA (Mahnashi et al. 2021; Silva et al. 2022). Our results show that GAL exerts promising effects against CFA-induced testicular injury by decreasing IL-6 and NF-κBp65 while increasing IL-10 expression. Notably, GAL was revealed to exert significant anti-inflammatory effects by downregulating pro-inflammatory signals in RA model slowing the destruction of the joints (Bartikoski et al. 2021; Gowayed et al. 2015), in a model of endotoxemia through reducing TNF-α (Pavlov et al. 2009), in a colitis model through reducing NF-κB, TNF-α, and RAGE while increasing IL-10 (Wazea et al. 2018), and in a model of Alzheimer's by reducing NF-κB (Joseph et al. 2020). Earlier studies on donepezil, an AchEI, reported its anti-inflammatory effect by reducing *p*-NF-κB in a model of LPS-induced neuroinflammation in rats (Kim et al. 2021).

Apoptosis is a genetically predetermined cell death that often occurs through intrinsic or extrinsic pathways and has a central role in testicular injury. Most frequently, these two routes of apoptosis are triggered by caspase activation that ends with executioner caspase-3 activation, which is the final indication of cell death (Saad et al. 2020). Pro-inflammatory cytokines and NF-κB p65 can induce apoptotic cell death by stimulating the caspase family (Luo et al. 2017). In addition, the attachment of germ cells to the seminiferous tubule epithelium requires testosterone; any decline in intra-testicular testosterone leads to the separation of seminiferous epithelium from germ cells, which would then induces germ cell death (Usende et al. 2021).

Our results demonstrated upregulation of apoptosis hallmark, caspase-3, in CFA-induced testicular injury. These results are consistent with earlier research that reported CFA increased expression of cleaved caspase-3 in an inflammation model (Baniasadi et al. 2020) and a model of pain-related recognition memory impairment (Rahmani et al. 2022). Enhanced apoptosis with consequent testes damage was reported in testicular injury in patients (Zhang et al. 2019). GAL significantly decreased caspase-3 expression in testicular damage by CFA, as revealed by immunohistochemical analysis. In line with these data, GAL counteracted apoptosis via caspase-3 inhibition in an Alzheimer’s model (El-Ganainy et al. 2022) and a model of myocardial ischemia–reperfusion in rats (Zeng et al. 2020b). Similarly, rivastigmine, an AchEI, exerted anti-apoptotic effects via downregulating caspase-3 levels in a model of RA in rats (Shafiey et al. 2018). These data provide evidence that GAL exerts potential anti-inflammatory and anti-apoptotic effects.

It was proved that IL-6 activates STAT3 by a JAK-dependent mechanism (Planas et al. 2006). JAK/STAT3 pathway activation is potentially involved in both cell proliferation and apoptosis. In the majority of cell types, STAT3 is crucial in controlling gene expression, cytokine responsiveness, and apoptotic cell death. In RA, phosphorylated STAT3 induces NF-ҡB and IL-6 expression in macrophages (Wang et al. 2012) causing impairment of sperm and reduced testosterone production. In addition, activation of the JAK/STAT3 signaling was proven to perform a crucial function in testicular injury pathogenesis (Hassanein et al. 2021). The JAK/STAT signaling is downstream for various ligand binding, including cytokines and ILs, to receptors on the cell surface causing dimerization of the receptor activating JAKs phosphorylation that motivate STATs in the cytosol via phosphorylation, causing STATs dimerization. These dimers enter the nucleus, bind to DNA-recognition motif consensus termed gamma-activated sites (GAS) in the promoter region of cytokine-inducible genes, and regulate the transcription of target genes encoding pro-inflammatory cytokines and chemokine (El-Gaphar et al. 2018).

SOCS3 plays a negative regulatory role in JAK/STAT3 signaling by inhibiting JAK kinase binding with receptors causing suppression of JAK phosphorylation to restrain STAT3 phosphorylation via Killer inhibitory receptor (Mahmoud and Abd El-Twab 2017; Oyinbo 2011). In experimental asthma model, α7nAChR exerted promising effects by inhibiting NF-κB/STAT3/SOCS3 signaling (Santana et al. 2017). Similarly, α7nAChR reduced the inflammatory response by modulating NF-κB (p65) and IL-6/JAK2/STAT3/SOCS3 signals in acute kidney injury (AKI) model (Ibrahim et al. 2018). Consequently, we aimed to explore the possible modulation of JAK/STAT3/SOCS3 signaling as a molecular mechanism for GAL protective effect. The current data show that CFA significantly enhanced testicular JAK and STAT3 phosphorylation with a substantial reduction in SOCS3 compared to control suggesting that this signaling pathway plays a main role in testicular injury by CFA (Alves-Silva et al. 2021)**.** In agreement with these results, a previous study revealed that CFA caused an increase in JAK/STAT3 protein phosphorylation in the model of arthritis (Soliman et al. 2022) and interstitial lung (Yang et al. 2019) while decreasing SOCS3 in arthritis model (Srivastava et al. 2022). Our findings showed that GAL successfully suppressed JAK/STAT3 while upregulated SOCS3 expression. Similar outcomes indicated the ability of GAL to abate the expression of JAK/STAT3 in a model of colitis (Wazea et al. 2018), high-fat diet (Ashmawy et al. 2022), and AKI (Ibrahim et al. 2018) while improving SOCS3 expression in inflammatory bowel disease (Seyedabadi et al. 2018). For the first time, the study's findings point to the beneficial effects of GAL against testicular injury by reducing the expression of JAK/STAT3/SOCS3 in testes tissue.

## Conclusion

**Fig. 8:**
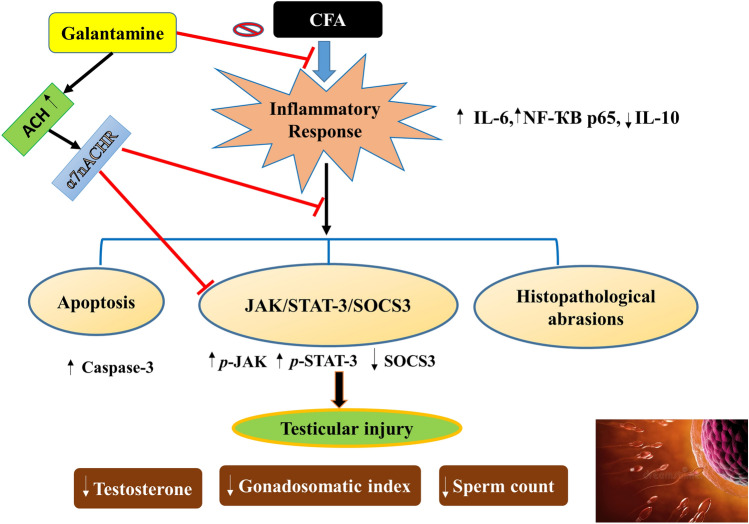
Diagram depicting mechanisms proposed for the protective effect of galantamine against testicular injury by CFA

Finally, this work suggests that using the anti-Alzheimer’s drug, GAL, could have a potential effect on testicular injury secondary to RA. GAL exerted potential protective effects against CFA-induced testicular damage through counteracting inflammation and apoptosis. GAL stimulated steroidogenesis by improving testosterone, sperm count, and gonadosomatic index and modulated JAK/STAT3/SOCS3 signaling. Additional clinical research is required to determine the effectiveness of GAL in treating testicular injury in RA-affected human individuals (Fig. 8).

## Data Availability

Data supporting the current results are available within the article or supplementary material.
